# Pyrotinib-based therapeutic approaches for HER2-positive breast cancer: the time is now

**DOI:** 10.1186/s13058-023-01694-5

**Published:** 2023-10-03

**Authors:** Xiaowei Qi, Qiyun Shi, Juncheng Xuhong, Yi Zhang, Jun Jiang

**Affiliations:** 1 Department of Breast and Thyroid Surgery, Southwest Hospital, Army Medical University, Chongqing, 400038 China; 2 The Eighth Medical Center of Chinese PLA General Hospital, Beijing, 100091 China; 3 Shigatse Branch, Xinqiao Hospital, Army Medical University, Shigatse, 857000 China

**Keywords:** Pyrotinib, HER-2 positive, Breast cancer, Targeted therapy, Tyrosine-kinase inhibitor

## Abstract

Human epidermal growth factor receptor 2 (HER2)-positive breast cancer (BC) is a highly aggressive subtype associated with poor prognosis. The advent of HER2-targeted drugs, including monoclonal antibodies, tyrosine-kinase inhibitors (TKIs) and antibody–drug conjugates, has yielded improved prognosis for patients. Compared with widely used monoclonal antibodies, small-molecule TKIs have unique advantages including oral administration and favorable penetration of blood–brain barrier for brain metastatic BC, and reduced cardiotoxicity. Pyrotinib is an irreversible TKI of the pan-ErbB receptor, and has recently been shown to be clinically effective for the treatment of HER2-positive BC in metastatic and neoadjuvant settings. This review highlights the development on the application of pyrotinib-based therapeutic approaches in the clinical settings of HER2-positive BC.

## Background

Breast cancer (BC) carries a high incidence and mortality in women worldwide [1]. Knowledge of BC pathogenesis and drug development has advanced and treatment strategies have improved, which has yielded increased long-term survival for patients.

BC is classified into four types based on molecular typing: luminal A, luminal B, human epidermal growth factor receptor 2 (HER2)-positive, and triple-negative [2, 3]. Among all types, HER2-positive BC accounts for approximately 15–20% of cases, and has highly aggressive biological properties [4].

HER2-targeted drugs have dominated treatment of HER2-positive BC [5]. Anti-HER2 drugs can be divided into three major categories: monoclonal antibodies (e.g., trastuzumab, pertuzumab, margetuximab, inetetamab), small-molecule tyrosine-kinase inhibitors (TKIs; e.g., pyrotinib, lapatinib, neratinib, tucatinib), and antibody–drug conjugates (ADCs: e.g., ado-trastuzumab emtansine [T-DM1], trastuzumab deruxtecan [T-DXd], disitamab vedotin [RC-48]). The advent of such drugs has provided more choices and chances for patients with HER2-positive BC [6].

## Overview of HER2 signaling pathways and anti-HER2 drugs

**Fig. 1:**
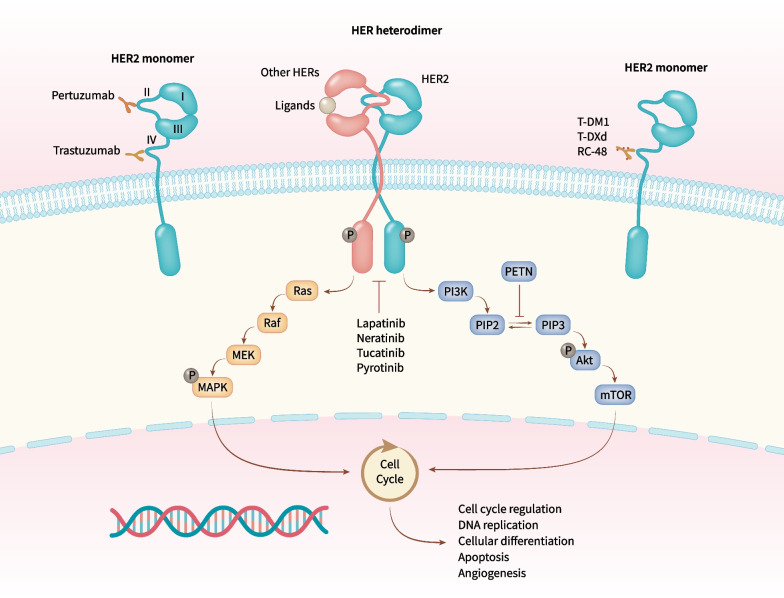
Role of HER2-targeted drugs in HER signaling. The four HER family receptors share structural homology with a structure comprising extracellular, transmembrane, and intracellular domains. The extracellular region comprised of four subdomains (I–IV) involved in ligand binding and receptor dimerization; the intracellular region was linked to the single-pass, hydrophobic transmembrane domain, comprising of tyrosine-kinase domain and a tail region that contains several sites of tyrosine phosphorylation. Of note, HER2 harbors no ligand-binding cleft and HER3 has defective intracellular kinase domain. HER2 can form homodimers and also heterodimers with HER-1, -3, and -4 in an open active conformation to regulate downstream signaling pathways, notably phosphoinositide 3-kinase/protein kinase B (PI3K/Akt) and Ras/mitogen-activated protein kinase (MAPK) pathways

The HER (also known as ErbB) family consists of types 1–4, with a structure comprising extracellular, transmembrane, and intracellular domains. HER1 and HER4 have a receptor-dependent tyrosine-kinase domain. HER2 contains a receptor-independent tyrosine-kinase domain. HER3 lacks a tyrosine-kinase domain [7]. HER-1, -3, and -4 bind to ligands via the extracellular domain to elicit conformational changes that expose their dimerization domain. HER2, independent of ligands, can form homodimers and also heterodimers with HER-1, -3, and -4 in an open active conformation to regulate downstream signaling pathways (e.g., phosphoinositide 3-kinase/protein kinase B [PI3K/Akt], Ras/mitogen-activated protein kinase [MAPK]), thereby affecting the proliferation and apoptosis of cells (Fig. 1) [8–10].

Trastuzumab is a humanized immunoglobulin G1 antibody. It can target the extracellular domain IV of HER2, blocking its ligand-independent activation and downstream signaling pathways. These actions have regulatory effects on the proliferation and apoptosis of tumor cells, as well as antibody-dependent toxicity to HER2-overexpressed cells [11, 12]. Pertuzumab targets the extracellular domain II of HER2, can inhibit the heterodimerization of HER2 with HER-1, -3, and -4, block downstream signaling pathways, and regulate the proliferation and apoptosis of tumor cells (Fig. 1) [13, 14]. Trastuzumab and pertuzumab have demonstrated good efficacy for treatment of HER2-positive BC in clinical trials (CLEOPATRA, PUFFIN, NeoSphere, PEONY) [15–18]. However, 10–20% patients achieve no benefits due to resistance to the effects of trastuzumab (approximately one-third have primary resistance and two-thirds have secondary resistance) and different types of drug resistance are driven by different mechanisms [19]. The main mechanism of primary resistance is that the extracellular target receptors of HER2 are inactivated and thereby lack binding sites for trastuzumab, so downstream PI3K/Akt/mammalian target of rapamycin (mTOR) signal transduction is blocked [19, 20]. The mechanisms of secondary resistance mainly involve: cluster of differentiation (CD)44^+^/CD24^−^ BC stem cells inhibiting the binding of trastuzumab to the extracellular domain of HER2 [21, 22]; signal masking by mucin-1 and mucin-4 [23, 24]; increased insulin-like growth factor I receptor signaling [25, 26]; altered beta-2 adrenergic receptor signaling [27]; blockade of phosphatase and tensin homolog (PETN)/PI3K/Akt signaling [28]; caveolae-mediated endocytosis [29]; cell-cycle changes that influence HER2 signaling [30].

Clinical data suggest that ADC and small-molecule TKIs could be solutions to the resistance of HER2-targeted antibody drugs. TKIs can compete for the intracellular adenosine triphosphate (ATP)-binding region of the HER family to form an ATP-like structure. In this way, TKIs can inhibit the phosphorylation of tyrosine kinases, block the transduction of downstream signaling pathways, and thereby suppress the growth of tumor cells. Clinical evidence has demonstrated the significant efficacy of TKIs such as lapatinib, neratinib, and tucatinib in patients with HER2-positive BC [31–34]. Moreover, for patients with brain metastases, if monoclonal antibody drugs cannot cross the blood–brain barrier (BBB), then small-molecule TKIs can cross the BBB to achieve better therapeutic effects [35–38]. In addition, TKIs have oral dosage forms, multiple targets, and low toxicity.

Pyrotinib is an irreversible TKI of the pan-ErbB receptor. By binding covalently to the ATP-binding site of the intracellular kinase domain of HER, pyrotinib can inhibit the autophosphorylation of the homodimers/heterodimers of HER, thereby blocking the Ras/Raf/MEK/MAPK and PI3K/Akt signaling pathways. The binding model of pyrotinib with the kinase domain of HER2 suggests that they are connected by a hydrogen bond between the *N*^1^ atom of 3-cyanoquinoline and hinge region Met-801, and that an irreversible covalent double bond is present between the inhibitor and Cys-805 through the Michael addition reaction. This scenario affects downstream signaling and prevents the development and progression of tumors [39, 40]. A phase-Ib clinical trial of pyrotinib monotherapy for advanced breast cancer (ABC) revealed that the maximum tolerated dose was 400 mg/day; pyrotinib (p.o.) could be absorbed completely within 1 h, reach a maximum plasma concentration after 4 h, and achieve a stable plasma concentration after 8 days of administration [41, 42]. In a phase II trial of pyrotinib or lapatinib combined with capecitabine for HER2-positive ABC, the independent radiologic committee-assessed objective response rate (ORR) was 71.4% in the pyrotinib group and 49.2% in the control group, and overall progression-free survival (PFS) was 18.1 months in the pyrotinib group and 7.0 months in the control group (a reduction in the risk of disease progression: 64%) [43]. Studies on use of pyrotinib for treatment of HER2-positive BC are discussed further below.

## Clinical evidence of pyrotinib in ABC

### First-line therapy for ABC

CLEOPATRA and PUFFIN trials established trastuzumab plus pertuzumab to be first-line treatment for ABC [15, 16]. However, only ~ 11% of patients had been treated previously with trastuzumab in either trial, which differs from current clinical practice. Considering that trastuzumab and/or pertuzumab has been used frequently in the neoadjuvant/adjuvant setting, whether the TKI pyrotinib (with its unique molecular structure and mechanism of action) can provide more benefits for such patients merits investigation.

Recently, the European Society for Medical Oncology published the results of the PHILA study on the efficacy and safety of pyrotinib or placebo combined with trastuzumab and docetaxel as first-line therapy for 590 patients with HER2-positive recurrent/metastatic BC. Investigator-assessed median PFS was 24.3 months and 10.4 months for the two groups, respectively; the proportions of patients treated previously with trastuzumab were 15.5% and 14.3%, respectively; subgroup analysis revealed that median PFS was not reached and was 9.3 months for patients with previous trastuzumab therapy, respectively, and 21.9 months and 10.4 months for those without previous trastuzumab therapy, respectively [44]. In a pooled analysis of three randomized controlled trials on pyrotinib (NCT02422199, NCT03080805, NCT02973737) involving a total of 145 female patients who received pyrotinib as first-line treatment for ABC, blinded independent central review-assessed median PFS was 12.4 months, and ORR was 72.4%; 89.0% patients had used trastuzumab previously, with a median PFS of 12.5 months, which was similar to the whole cohort [45]. The PANDORA trial (NCT03876587) revealed favorable efficacy of pyrotinib plus docetaxel as first-line therapy for HER2-positive metastatic BC. Seventy-nine patients were enrolled, of whom 65 could be evaluated. ORR was 78.5% for 65 patients, 83.3% for patients with previous trastuzumab treatment (accounting for 30.4%), 74.5% for those without previous trastuzumab treatment (accounting for 68.6%), 89.5% for those with visceral metastases, and 73.3% for those without visceral metastases [46]. Those studies demonstrated the promising efficacy of pyrotinib as first-line therapy for HER2-positive ABC regardless of previous use of trastuzumab.

### Second-line therapy for ABC

The PHOEBE trial assigned 267 patients to receive pyrotinib plus capecitabine or lapatinib plus capecitabine. Median PFS was 12.5 months and 6.8 months, respectively (hazard ratio [HR] = 0.39, 95% confidence interval [CI] 0.27–0.56, *P* < 0.0001). Median overall survival (OS) was not reached and was 26.9 months, respectively (HR = 0.69, 95% CI 0.48–0.98, *P* = 0.02). Subgroup analysis revealed significant benefits regardless of previous use of trastuzumab: median PFS was 12.5 months and 6.9 months for patients with previous trastuzumab treatment, respectively; median PFS was 12.5 months and 5.6 months for patients who had used trastuzumab before, respectively; OS was not reached [47, 48].

The PHENIX trial investigated the efficacy of pyrotinib plus capecitabine *versus* placebo plus capecitabine for patients who had had disease progression during or after trastuzumab treatment or who could not receive trastuzumab or lapatinib. Independent review committee-assessed median PFS was 11.1 months and 4.1 months, respectively (HR = 0.18, 95% CI 0.13–0.26, *P* < 0.001). In terms of secondary endpoints: ORR was 68.6% and 16.0%, respectively; clinical benefit was achieved in 76.8% and 22.3% of cases, respectively; median OS was 34.9 months and 23.6 months, respectively (HR = 0.74, 95% CI 0.54–1.02, *P* = 0.068). Subgroup analysis demonstrated that pyrotinib plus capecitabine was significantly superior to placebo plus capecitabine regardless of metastatic sites or the status of the hormone receptor and trastuzumab resistance [49, 50].

Pyrotinib exhibits superior effects in prolonging PFS to other types of second-line therapy for ABC. Median PFS has been reported to be 9.6 months using T-DM1 alone [51], 8.4 months using lapatinib plus capecitabine [52], 8.2 months using trastuzumab plus capecitabine [53], and 2.8 months using trastuzumab plus lapatinib [54]. Multiple drugs have been approved for second-line therapy, but availability between countries/regions differs. Based on efficacy and safety evidence, pyrotinib has been recommended as preferred second-line therapy in Chinese clinical guidelines [55, 56].

### Third-/later-line therapy for ABC

Third-/later-line treatment of ABC is complicated. Most patients develop drug resistance after experiencing various types of therapy (e.g., targeted, endocrine, chemotherapy), accompanied by multiple metastases. Treatment strategies should be formulated based on comprehensive factors.

**Fig. 2:**
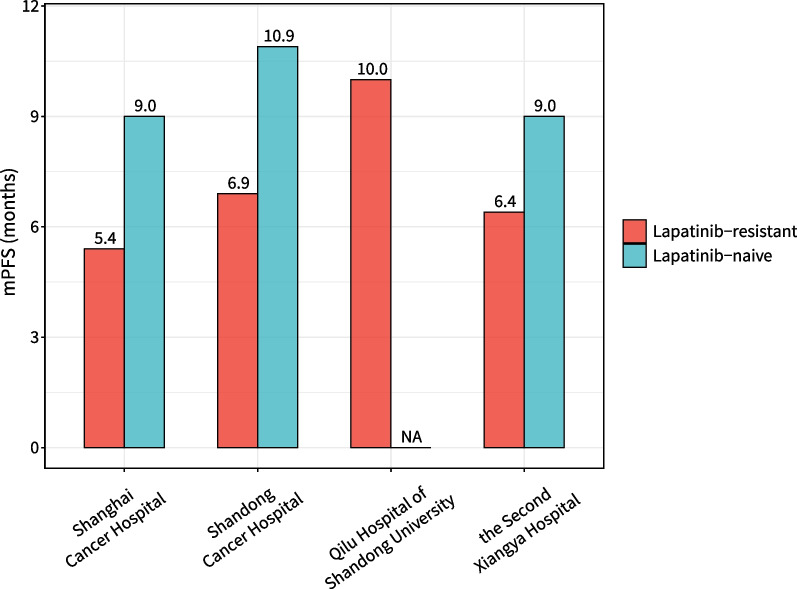
Median PFS of lapatinib-resistant and -naive patients after later-line treatment with pyrotinib in advanced stage (mPFS, median progression-free survival; NA, not applicable)

A real-world study evaluated the efficacy of pyrotinib plus capecitabine *versus* trastuzumab plus capecitabine as second-/later-line anti-HER2 therapy for patients with ABC: compared with the trastuzumab group (100 patients), the pyrotinib group (81 patients) showed significantly higher ORR (42.00% vs. 58.02%, *P* = 0.037) and significantly longer median PFS (7.11 months vs. 8.02 months, *P* = 0.035) [57]. In a real-world study investigating the efficacy of pyrotinib in the setting of lapatinib resistance (most patients had been treated with ≥ 2 lines of anti-HER2 regimens), 113 patients were assigned to receive a combination of pyrotinib plus capecitabine, vinorelbine, or trastuzumab; median PFS was 5.4 months for lapatinib-resistant patients and 9 months for lapatinib-naive patients [58]. Sun et al. [59] reported that, among 64 patients with ABC who had received multiple lines of treatment, 17.2% were resistant to lapatinib, with an ORR of 44.1% and a median PFS of ~ 10 months, after pyrotinib-based therapy. In a real-world study involving 94 patients (31.9% with resistance to lapatinib), for lapatinib-resistant and lapatinib-naive patients, pyrotinib-based treatment generated median PFS of 6.36 months and 9.02 months and median OS of 14.35 months and 20.73 months, respectively [60]. Another real-world study involving 218 patients (40.8% with previous use of lapatinib) showed that median PFS was 6.8 months with pyrotinib-based therapy as third-line treatment [61]. Those studies indicated that pyrotinib showed encouraging efficacy even after failure of multiple lines of therapy (Fig. 2).

### Brain metastases

Patients with HER2-positive ABC are at high risk of developing brain metastases, which confers a poor prognosis [62]. In addition to local treatment, efficacious systemic treatment is vital for resolving brain metastases. The PERMEATE trial involving 78 patients with HER2-positive BC with brain metastases revealed that, for radiotherapy-naive and radiotherapy-resistant cohorts receiving pyrotinib plus capecitabine, the intracranial ORRs were 74.6% (95% CI 61.6–85.0) and 42.1% (95% CI 20.3–66.5), respectively, and median PFS was 11.3 months (95% CI 7.7–14.6) and 5.6 months (95% CI 3.4–10.0), respectively. Also, the most common adverse events of grade ≥ 3 were diarrhea (24%), reduced white blood cell count (14%), and reduced neutrophil count (14%), which were (in general) manageable [63].

Real-world studies have also demonstrated the stable and reliable efficacy of pyrotinib in patients with brain metastases [64–66]. In a real-world study of 113 patients, 31 patients with brain metastases receiving pyrotinib-containing treatment showed a median PFS of 6.7 months and an intracranial ORR of 28% [58]. Another real-world study reported various efficacy indicators of pyrotinib-based therapy in 42 patients with ABC suffering from brain metastases. ORR was 40.4%, disease control was obtained in 92.8% of cases, improvement in intracranial symptoms was noted in all patients, the median duration of intracranial improvement was 15 months, the median time to relieve brain metastases was 43 days, the median time to relieve other metastases was 50 days, the median time to progression of brain metastases was 16.6 months, and the median time to disease progression was 11.1 months [67]. In a retrospective study involving 61 HER2-positive patients with brain metastases treated by pyrotinib-based regimens, median PFS was 8.6 months, median OS was 18.0 months, and the combination of pyrotinib with nab-paclitaxel was superior to the combination with capecitabine and vinorelbine with respect to PFS and OS. Those studies suggested that the unique structure and low molecular weight of pyrotinib enabled BBB crossing, thereby generating favorable therapeutic effects upon brain metastases. Ongoing research may provide more evidence for the therapeutic value of pyrotinib in patients with ABC with brain metastases, and further optimize the use of pyrotinib.

## Clinical evidence of pyrotinib in early BC

### Neoadjuvant therapy

According to guidelines set by the National Comprehensive Cancer Network in 2022 and American Society of Clinical Oncology in 2021 [68, 69], neoadjuvant therapy is recommended for patients with HER2-positive BC with tumor size > 2 cm and/or a positive lymph node status (LN+). Neoadjuvant therapy for HER2-positive BC has evolved from single trastuzumab targeting to trastuzumab-based dual targeting. The NOAH study established the role of single-target drugs in neoadjuvant therapy for HER2-positive BC [70]. In NeoSphere and PEONY studies, total pathologic complete response (tpCR) with trastuzumab plus pertuzumab was achieved in 39.3% of cases, which was significantly superior to that of single-target therapy and chemotherapy [17, 18]. Small-molecule TKIs and macromolecule monoclonal antibodies act on intracellular and extracellular target sites simultaneously to exhibit synergistic anti-HER2 effects. The NeoALTTO trial assigned 455 patients to receive trastuzumab plus lapatinib, lapatinib alone, or trastuzumab alone, and pathologic complete response (pCR) was achieved in 51.3%, 24.7%, and 29.5% of cases, respectively, which demonstrated the superior efficacy of trastuzumab plus TKI in the neoadjuvant setting [33]. A meta-analysis of 1410 patients (from CALGB 40601, CHER-LOB, NSABP-B41, and NeoALTTO trials) revealed that, compared with trastuzumab monotherapy, lapatinib plus trastuzumab improved recurrence-free survival significantly (HR = 0.62, 95% CI 0.46–0.85) and OS (HR = 0.65, 95% CI 0.43–0.98) upon combination with neoadjuvant chemotherapy [71]. Those results indicated that a combination of trastuzumab with TKIs could be a promising neoadjuvant strategy.

**Fig. 3:**
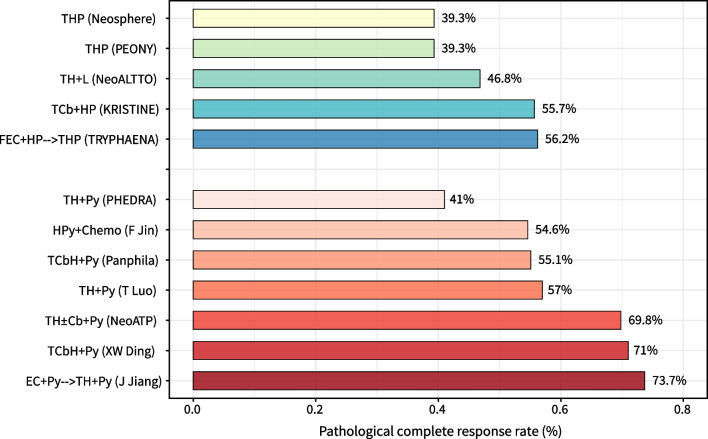
Comparison of pathologic complete response rate between neoadjuvant chemotherapy regimens from different clinical studies (T, taxanes; H, trastuzumab; P, pertuzumab; L, lapatinib; Cb, carboplatin; F, fluorouracil; E, epirubicin; Py, pyrotinib; Chemo, chemotherapy)

We researched the use of pyrotinib in neoadjuvant therapy for HER2-positive BC: 19 patients received four cycles of ECP (epirubicin, cyclophosphamide, pyrotinib) and then four cycles of THP (docetaxel, trastuzumab, pyrotinib) before surgery, and tpCR was achieved in 73.7% (95% CI 48.8–90.9), and ORR was 100% (95% CI 82.4–100) of cases [72]. Subsequent clinical trials confirmed the favorable activity of pyrotinib in neoadjuvant therapy. In the PHEDRA trial (NCT03588091), 355 patients were assigned randomly to receive pyrotinib or placebo in combination with trastuzumab and docetaxel for four cycles before surgery; the pyrotinib group showed a significantly higher rates of tpCR (41.0% vs. 22.0%) and breast pCR (43.8% vs. 23.7%) (assessed by an independent review committee) than the placebo group [73]. The multicenter phase II Panphila trial reported a pCR rate of 55.1% in 69 patients with HER2-positive BC receiving six cycles of neoadjuvant therapy with TCbHPy (docetaxel, carboplatin, trastuzumab, pyrotinib) [74]. In the phase II NeoATP trial, the pCR rate reached 69.81% in 53 patients with HER2-positive local ABC (stage IIA–IIIC) receiving four cycles of pyrotinib plus trastuzumab and paclitaxel-cisplatin as neoadjuvant treatment [75]. A retrospective study of 545 patients revealed that in the neoadjuvant setting, the pCR rate with TCbHPy was superior to that with TCbH and comparable to that with TCbHP (docetaxel, carboplatin, trastuzumab, pertuzumab) in HER2-positive local ABC [76]. Those results demonstrated that pyrotinib could significantly improve the pCR and ORR of patients under neoadjuvant treatment (Fig. 3), thereby increasing the possibility of rapid tumor shrinkage and cure at an early stage. As shown in the studies stated above, chemotherapy regimens in combination with trastuzumab and pyrotinib vary. Optimizing chemotherapy combinations and balancing neoadjuvant efficacy and toxicity are key problems to be explored further. Clinical studies on neoadjuvant therapy using pyrotinib are summarized in Table 1.

**Table 1:** Clinical studies on neoadjuvant therapy using pyrotinib

No.	Registration ID	Title	Target sample size	Outcomes
1	ChiCTR2100052892	Pyrotinib as neoadjuvant treatment for HER2-positive breast cancer: a single-arm, multicenter, prospective observational trial	100	pCR, EFS, DFS, DMFS, ORR
2	ChiCTR2100048136	Trastuzumab combined with pertuzumab and sequential use of pyrotinib vs trastuzumab combined with pertuzumab for adjuvant treatment of non-pCR HER2-positive breast cancer after neoadjuvant therapy: a prospective, randomized control, stage iii clinical trial	450	iDFS, DFS, OS, DDFS
3	ChiCTR2100047086	Single-Arm, Multicenter Clinical Study of Pyrotinib Maleate Tablets Combined with Albumin-Bound Paclitaxel and Trastuzumab in Neoadjuvant Treatment of Her2-positive Early or Locally Advanced Breast Cancer	199	pCR, ORR, DCR, bpCR, safety
4	ChiCTR2000034827	Pyrotinib maleate, CDK4/6 inhibitor and letrozole in combination for treatment of stage II–III triple-positive breast cancer: a phase II clinical trial	89	tpCR, BORR, RCB, OS, DFS
5	ChiCTR1900028212	A multicenter, prospective, single-arm, exploratory clinical study of neoadjuvant therapy of her2-positive breast cancer with pyrotinib maleate tablets combined with paclitaxel for injection (albumin-bound)	90	pCR, EFS, DFS, DDFS, ORR safety
6	ChiCTR1900026200	Neoadjuvant chemotherapy with pyrotinib, trastuzumab, docetaxel, and carboplatin in combination for locally advanced epidermal growth factor receptor 2-positve breast cancer: a multicenter, randomized, open-label, parallel-group controlled phase III trial	532	tpCR, ORR, ECOG PS, safety
7	ChiCTR1800020226	Prospective, open-label, multicenter trial for pyrotinib plus trastuzumab, carboplatin, and docetaxel in the treatment of HER2-positive breast cancer	236	tpCR, EFS, DFS, ORR, DDFS
8	ChiCTR2200062936	Pyrotinib as neoadjuvant treatment for HER2-positive breast cancer	300	pCR, DMFS, ORR, OS, safety
9	NCT04917900	Single-arm, Multicenter Clinical Study of Pyrotinib Maleate Tablets Combined with Albumin-bound Paclitaxel and Trastuzumab in Neoadjuvant Treatment of HER2-positive Early or Locally Advanced Breast Cancer	199	pCR, ORR, DCR, bpCR, AEs
10	NCT04900311	Pyrotinib Versus Pertuzumab in Combination with Neoadjuvant Trastuzumab and Nab-Paclitaxel in HER2+ Early or Locally Advanced Breast Cancer	490	tpCR, iDFS, EFS, ORR, BCS rate
11	NCT03847818	Neoadjuvant Study of Pyrotinib and Trastuzumab Plus Chemotherapy in Patients with HER2 Positive Breast Cancer	268	pCR, EFS, DFS, DDFS, ORR
12	NCT04872985	Pyrotinib in Combination with Neoadjuvant Chemotherapy in HR+ /HER2−, HER4 High Expression Breast Cancer Patients: A Phase II Trial	140	tpCR, pCR, ORR, EFS, OS
13	NCT04398914	Pyrotinib, Trastuzumab, Pertuzumab and Nab-paclitaxel as Neoadjuvant Therapy in HER2-positive Breast Cancer	216	tpCR, bpCR, EFS, DFS, OS
14	NCT03588091	Neoadjuvant Study of Pyrotinib in Combination with Trastuzumab in Patients with HER2 Positive Breast Cancer	355	pCR evaluated by IRC, pCR evaluated by sites, EFS, DFS, DDFS, ORR
15	NCT03756064	Neoadjuvant Study of Pyrotinib in Patients with HER2 Positive Breast Cancer	100	pCR, EFS, DFS, DDFS, ORR
16	NCT04290793	Neoadjuvant Chemotherapy with Pyrotinib, Epirubicin and Cyclophosphamide Followed by Taxanes and Trastuzumab for HER2+ Breast Cancer	280	pCR, ORR, EFS, DFS, OS
17	NCT05561686	Real-world Study of Pyrotinib in Neoadjuvant Therapy for HER2-positive Breast Cancer	100	tpCR, bpCR, ORR, AEs
18	NCT05426486	A Randomized, Open-Label, Multicenter Phase II–III Neoadjuvant Study Comparing the Efficacy and Safety of ARX788 Combined with Pyrotinib Maleate Versus TCBHP (Trastuzumab Plus Pertuzumab with Docetaxel and Carboplatin) in Patients with HER2-positive Breast Cancer	150	tpCR, bpCR, RCB, BORR, OS, DFS, AEs

*iDFS* invasive disease-free survival; *DFS* disease-free survival; *OS* overall survival; *DDFS* distant disease-free survival; *pCR* pathologic complete response rate; *ORR* objective response rate; *DCR* disease control rate; *bpCR* breast pathologic complete response rate; *BORR* best overall response rate; *RCB* residual cancer burden; *EFS* event-free survival; *ECOG PS* Eastern Cooperative Oncology Group performance status; *DMFS* distant metastasis-free survival; *AEs* adverse events; *BCS rate* the rate of adopting breast-conserving surgery; *IRC* independent review committee

### Adjuvant therapy

Since failure of the ALLTO trial [77], few studies have investigated the efficacy of adjuvant TKIs for HER2-positive BC. The BCIRG006, NSABP B-31/NCCTG N9831, and HERA studies demonstrated that trastuzumab administered in the adjuvant setting can control disease progression effectively [78–80]. The KATHERINE trial revealed that adjuvant T-DM1 greatly increased the 3-year invasive disease-free survival (iDFS) rate of patients with HER2-positive BC who did not achieve pCR who had received neoadjuvant therapy. In the APHINITY trial, pertuzumab plus trastuzumab with chemotherapy significantly increased the 6-year iDFS rate compared with trastuzumab with chemotherapy, especially for LN+ patients [81]. The ExteNET trial is the only one with successful results with TKIs in the adjuvant setting. That study randomly assigned 2840 patients treated with adjuvant trastuzumab and chemotherapy to receive neratinib or placebo for 1 year; compared with placebo, neratinib increased the 5-year iDFS rate significantly by 2.5% (87.7% vs. 90.2%) and by 3.7% (86.6% vs. 91.2%) in the LN+ subgroup analysis [82]. Whether pyrotinib can be used in intensive adjuvant therapy, especially for high-risk patients (LN+, non-pCR), merits attention. An ongoing phase III trial (CTR20191261) is exploring extended adjuvant therapy (pyrotinib following trastuzumab) in LN+ patients who had been treated with trastuzumab and/or pertuzumab. That study could provide more data for adjuvant application of TKIs. Clinical studies on adjuvant pyrotinib therapy are summarized in Table 2.

**Table 2:** Clinical studies on adjuvant therapy using pyrotinib

No.	Registration ID	Title	Target sample size	Outcomes
1	ChiCTR2200058746	Multicenter cohort study on efficacy and safety of HER2-positive, node-positive breast cancer following intensive adjuvant or neoadjuvant anti-HER2 therapy with pyrotinib	200	iDFS, DDFS, OS
2	ChiCTR2100049018	A randomized, open-label, multicenter study to evaluate the efficacy and safety of continuation of original targeted therapy versus Trastuzumab combined with Pyrotinib and capecitabine as postoperative adjuvant therapy in patients with HER2-positive early breast cancer who have residual tumor present pathologically following	206	3-year iDFS rate
3	ChiCTR2000040866	Comparison of Pyrotinib or Pertuzumab Combined with Trastuzumab for non-pCR HER2 Positive Breast Cancer after Neoadjuvant Therapy: A Randomized, Open, Prospective Clinical Study	546	iDFS, DFS, OS, BCSS, safty
4	ChiCTR2000038503	Pyrotinib and Trastuzumab for Early or Local Advanced HER2-Positive Breast Cancer	97	iDFS, OS, DFS, DDFS, safty
5	NCT04254263	Adjuvant Study of Pyrotinib in HER2 Positive Breast Cancer (ATP)	316	iDFS, DFS, OS
6	NCT04659499	Nab-paclitaxel in Combination with Pyrotinib in Postoperative Adjuvant Therapy for HER2-positive Breast Cancer	261	3-year DFS, AEs + SAEs
7	NCT03980054	A Study of Evaluating the Effects of Pyrotinib After Adjuvant Trastuzumab in Women with Early stage Breast Cancer	1192	iDFS, DFS, OS, DDFS
8	NCT05292742	Compare Continuation of Original Targeted Therapy with Trastuzumab Combined with Pyrotinib and Capecitabine as Postoperative Adjuvant Therapy in Non-pCR Patients with HER2 Positive Early Breast Cancer	206	iDFS

*iDFS* invasive disease-free survival; *DDFS* distant disease-free survival; *OS* overall survival; *DFS* disease-free survival; *BCSS* breast cancer-specific survival; *AEs* adverse events; *SAEs* severe adverse events

### Toxicity of pyrotinib and management

Owing to its unique structure and pharmacological mechanism of action, pyrotinib exhibits favorable efficacy and effective tumor control in HER2-positive BC but, simultaneously, its adverse reactions (e.g., diarrhea) trouble patients. In the PHOEBE, PHENIX, and PANDORA trials, the incidence rates of diarrhea of grade ≥ 3 were 31%, 33%, and 38.2%, respectively [46, 47, 49]. The PHADRA and PHILA trials also reported a high rate of diarrhea of grade ≥ 3 [44, 73]. Fortunately, this problem has some solution in intent-to-treat analysis. The PANDORA trial revealed that prophylaxis using loperamide reduced the incidence of diarrhea of grade ≥ 3 significantly from 38.2 to 8.9% [46]. ChiCTR2200060339 [83] and ChiCTR2100051163 [84] are also exploring active management of diarrhea to reduce diarrhea of grade ≥ 3. In clinical practice, to increase adherence and extend treatment cycles, the tolerability of pyrotinib can be improved by: establishing patients’ expectations of adverse reactions; reducing patients’ psychological burden such as fear; preventive treatment with loperamide; avoiding long-term diarrhea-induced negative conditions such as anorexia and fatigue.

### Biomarkers of pyrotinib efficacy

A phase-I clinical study reported that the efficacy of pyrotinib could be predicted by the levels of phosphatidylinositol-4,5-bisphosphate 3-kinase catalytic subunit alpha (PIK3CA) and TP53 mutations in circulating tumor DNA rather than in tumor cells [41]. The NeoATP study [75] revealed that pCR was more likely to be achieved in patients who were estrogen receptor-negative progesterone receptor-negative, HER2 3+ by immunohistochemistry (IHC), with a HER2/CEP17 ratio ≥ 4, and HER2 copy number ≥ 14. pCR was not related to PIK3CA status, Ki67 index, or stromal tumor-infiltrating lymphocytes (TILs). The Panphila study [74] confirmed that patients with hormone receptor-negative disease and HER2 IHC 3+ were more likely to achieve pCR. In addition, the pCR rate was independent of the TIL level regardless of whether the threshold of the TIL level was defined as 5% or 50%, and the TIL level was similar in pCR and non-pCR cohorts. Multiplex IHC results revealed associations of pCR with stromal levels of CD20, CD8, CD4, and forkhead box P3 (FOXP3) and epithelial levels of CD20, CD8, and CD4 before treatment. Among them, stromal levels of CD20, CD8, and CD4 and the epithelial level of CD8 were determined to be independent predictors of pCR according to multivariable logistic regression analysis. Based on stromal immune markers, unsupervised hierarchical clustering analysis revealed that patients with high levels of CD20, CD8, CD4, and FOXP3 simultaneously had a higher possibility of pCR. We assessed 425 genes in tumor samples from patients receiving neoadjuvant therapy with pyrotinib, trastuzumab, and chemotherapy. We concluded that the PIK3CA mutation was an independent predictor of therapeutic effects; patients with a PIK3CA mutation were less likely to achieve pCR, whereas the TIL level was not associated with pCR [85]. Those biomarker studies could preliminarily guide the selection of patients more likely to benefit from pyrotinib-based regimens. Ongoing biomarker studies may provide more information on the use of pyrotinib for BC.

## Conclusions

At present, among four approved anti-HER2 TKI drugs in China, pyrotinib has more robust clinical evidence and covers more people in clinical practice. Compared with lapatinib, PHOEBE study demonstrated that pyrotinib can significantly prolong PFS in metastatic setting [47]. In terms of neratinib, NEfERT-T trial failed to prove that neratinib–paclitaxel was superior to trastuzumab–paclitaxel in first-line HER2-positive ABC [37]. Compared with tucatinib, whose benefit is limited to metastatic setting, evidence supports clinical benefit of pyrotinib in both early and advanced stage.

Pyrotinib shows encouraging efficacy in neoadjuvant, advanced-stage, first-/second-/later-line, and brain-metastases settings, as well as in triple-positive patients. With excellent therapeutic effects, pyrotinib is changing the landscape of BC treatment. Future research should focus on how to select and identify patients who are more likely to benefit from pyrotinib-containing combinations. For example, does combination with pyrotinib have greater efficacy for patients who progress rapidly after (neo)adjuvant treatment with macromolecular antibodies such as trastuzumab? Can pyrotinib prevent and reduce the risk of metastases to the central nervous system? Why are patients sensitive or resistant to pyrotinib, and could the related molecular markers be identified? In which populations can combination with pyrotinib better compensate for the deficiency of macromolecular antibody drugs? Another focus is how to identify (at an early stage) patients prone to pyrotinib-related diarrhea and formulate strategies for optimal management of diarrhea, which can help deepen understanding of the toxicity of pyrotinib and improve its safety and patient adherence. Such explorations will help maximize the benefits of patients taking pyrotinib.

## Data Availability

Not applicable.

## Abbreviations

BC: Breast cancer
HER2: Human epidermal growth factor receptor 2
TKI: Tyrosine-kinase inhibitor
ADC: Antibody–drug conjugate
T-DM1: Ado-trastuzumab emtansine
PI3K: Phosphoinositide 3-kinase
CD: Cluster of differentiation
ATP: Adenosine triphosphate
BBB: Blood–brain barrier
MAPK: Mitogen-activated protein kinase
ABC: Advanced breast cancer
ORR: Objective response rate
PFS: Progression-free survival
HR: Hazard ratio
CI: Confidence interval
LN: Lymph node
tpCR: Total pathologic complete response
pCR: Pathologic complete response
iDFS: Invasive disease-free survival
PIK3CA: Phosphatidylinositol-4,5-bisphosphate 3-kinase catalytic subunit alpha
IHC: Immunohistochemistry
FOXP3: Orkhead box P3
